# Comparison of diagnostic performance between contrast-enhanced mammography and breast magnetic resonance imaging

**DOI:** 10.3389/fonc.2025.1557800

**Published:** 2026-01-08

**Authors:** Ying Yang, Xiangying Yang, Fan Yang

**Affiliations:** Department of Radiology, Union Hospital, Tongji Medical College, Huazhong University of Science and Technology, Wuhan, Hubei, China

**Keywords:** breast cancer, contrast-enhanced mammography, diagnostic performance, magnetic resonance imaging, mammography

## Abstract

**Objective:**

Contrast-enhanced mammography (CEM) is an emerging imaging technique. This study aims to compare the diagnostic accuracy of CEM and magnetic resonance imaging (MRI) for breast lesions.

**Methods:**

A retrospective analysis was conducted on patients who underwent both CEM and MRI at our institution from July 2019 to April 2024. All imaging reports were prepared in accordance with the Breast Imaging Reporting and Data System (BI-RADS) guidelines established by the American College of Radiology (ACR). The final diagnosis was established using pathology as the “gold standard.” The diagnostic performance of the two methods in detecting breast lesions was compared using sensitivity, specificity, accuracy, positive predictive value (PPV), negative predictive value (NPV), and receiver operating characteristic (ROC) curves.

**Results:**

A total of 292 patients underwent paired CEM and MRI examinations, with an average age of 46.9 years. Among the 301 breast lesions identified, 121 (40.2%) were benign, and 180 (59.8%) were malignant. The sensitivity, specificity, accuracy, positive predictive value, and negative predictive value of CEM for diagnosing benign and malignant breast lesions were 98.9%, 78.5%, 90.7%, 87.3%, and 97.9%, respectively. The corresponding values for MRI were 98.9%, 72.7%, 88.4%, 84.4%, and 97.8%. No significant difference was observed in the diagnostic performance between the two methods (p > 0.05). The areas under the curve (AUC) for CEM and MRI were 0.887 (95% CI: 0.85–0.92) and 0.858 (95% CI: 0.81–0.90), respectively (p = 0.400).

**Conclusion:**

As an emerging imaging technique for detecting and diagnosing breast lesions, CEM demonstrates diagnostic performance comparable to that of MRI.

## 1 Introduction

Breast cancer is a global public health issue and is currently the most common type of cancer (1). In China, breast cancer ranks first in terms of incidence among various malignant tumors in women (2). Therefore, early detection, early diagnosis, and early treatment are crucial for improving the quality of life and prolonging survival in breast cancer patients. The most commonly used methods currently include routine full-field digital mammography (FFDM), ultrasound, and MRI. However, FFDM images are susceptible to interference from the surrounding glandular tissue, which may obscure the lesions and cause overlapping of abnormal tissues, making it particularly challenging to distinguish lesions from dense glandular tissue, especially in Asian women, whose breast tissue tends to be denser. This can lead to missed or misdiagnosed cases. Studies show that the overall sensitivity of FFDM for breast cancer screening is approximately 79.9%, but for women with dense breasts, this sensitivity drops to about 50% (3). Another study reported that for women with entirely fatty breasts, the sensitivity of FFDM was 87.0% and specificity was 96.9%, while for women with extremely dense breasts, the sensitivity dropped to 62.9% and specificity to 89.1% (4). Ultrasound is a supplementary screening method for women with dense breasts, but its relatively higher false positive rate makes it less preferred by breast surgeons.

Currently, breast MRI is a sensitive examination method recommended by both domestic and international guidelines for preoperative evaluation of breast cancer. MRI plays a critical role in detecting and characterizing breast cancer lesions, assessing the local extent of the disease, evaluating treatment response, and guiding biopsy localization. Large-scale meta-analyses indicate that breast MRI has a sensitivity exceeding 90% for diagnosing malignant breast lesions (5). However, it also faces several clinical challenges. For example, MRI equipment and medical costs are relatively high, and imaging takes a longer time, leading to limited clinical accessibility. Additionally, for patients with contraindications to MRI (such as claustrophobia or specific implants like pacemakers or cochlear implants), alternative diagnostic methods are required. MRI also has some diagnostic limitations, including insensitivity to calcifications and a relatively high false positive rate. Different studies report significant variations in MRI specificity, ranging from 25% to 100% (6–8).

Contrast-enhanced mammography (CEM) is an emerging breast imaging technique that combines traditional mammography with contrast enhancement. By utilizing the K-edge effect of iodine, CEM involves intravenous injection of a contrast agent, followed by rapid high and low energy dual-energy exposure. After post-processing, low-energy and subtraction images are obtained (9, 10). CEM provides similar morphological information to FFDM on low-energy images and detailed calcification displays, while subtraction images can eliminate the overlap effect of glandular tissue, offering a more accurate reflection of the lesion’s hemodynamics and enhancing the visualization of tumor neovascularization, leading to a more precise assessment of the lesion size compared to pathology results (11). Compared to FFDM, CEM has higher sensitivity and specificity, improving lesion detection and diagnostic accuracy (12–14).

In China, the population with dense breast tissue is larger compared to Europe and the U.S. Therefore, CEM can offer more clinical benefits for Chinese patients. Furthermore, CEM is easy to perform, relatively inexpensive, has controllable radiation exposure, and has low technical diffusion difficulty, making it highly suitable for implementation in urban communities and rural healthcare settings. The purpose of this study is to explore and compare the diagnostic performance of CEM and MRI in the evaluation of breast diseases.

## 2 Materials and methods

### 2.1 Patients selection

**Figure 1:**
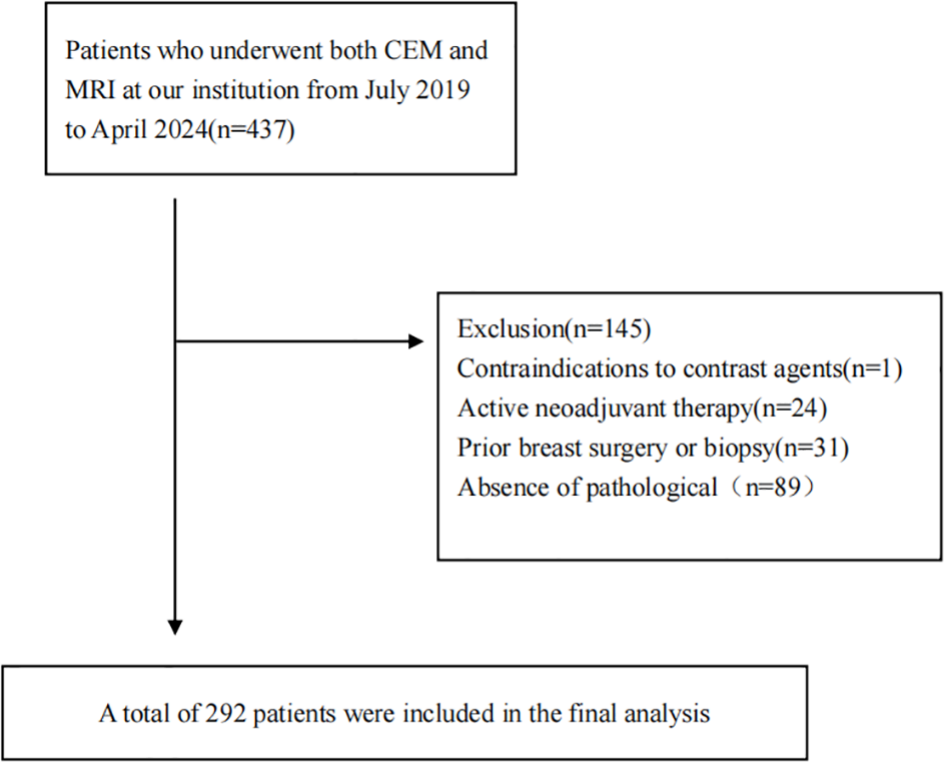
Flowchart of the study population.

This study protocol was approved by the Institutional Ethics Committee of our hospital. The requirement for informed consent was waived due to the retrospective nature of the study and the anonymized processing of all data. We retrospectively collected the imaging data of patients who underwent CEM and MRI examinations at our hospital between July 2019 and April 2024. Patient enrollment flowchart is detailed in **Figure 1**.

#### 2.1.1 Inclusion criteria

Patients suspected of having abnormal lesions based on clinical examination or breast screening;

Patients who underwent both MRI and CEM examinations within one week;

Patients whose final diagnosis was confirmed through biopsy or surgical pathology.

#### 2.1.2 Exclusion criteria

Contraindications to contrast agents, such as nephrotoxicity, allergic reactions, etc.;

Patients currently undergoing neoadjuvant chemotherapy, hormone therapy, or radiotherapy for this disease;

Patients who have already undergone any surgical diagnostic or therapeutic procedures for this disease, including biopsies or therapeutic breast surgeries;

No pathological biopsy was performed for the lesion.

### 2.2 Contrast-enhanced mammography

CEM was performed using the GE Senographe Essential full-field digital mammography machine. The contrast agent, iohexol (300–370 mg/ml), was injected into the radial vein at a dose of 1.5 ml/kg and a flow rate of 3.0 ml/s. After 2 minutes, the patient underwent craniocaudal (CC) and mediolateral oblique (MLO) mammography in both positions, simultaneously collecting low-energy(∼28–32 kVp) and high-energy(∼45–49 kVp) images. Subtraction images were subsequently generated automatically by the vendor’s software using a weighted log-subtraction algorithm to suppress the background breast tissue and enhance the contrast uptake areas. A total of 8 images were collected, including low-energy and subtraction images of the affected and unaffected sides in both CC and MLO views. After the examination, patients were observed for 30 minutes to ensure no obvious adverse reactions before being discharged.

### 2.3 Magnetic resonance imaging

MRI was performed using a 3.0T MRI scanner (Siemens Skyra) with a dedicated four-channel breast coil. The scanning sequence included TIRM (turbo inversion recovery magnitude) for the axial plane, T1-weighted fat-suppressed images, and diffusion-weighted imaging (DWI). After the unenhanced scan, gadolinium contrast agent (Gd-DTPA) was administered at a dose of 0.1 mmol/kg body weight via an automatic injector at a rate of 2.0 ml/s. A 20 ml saline flush followed the injection. The enhanced scan was conducted with fat suppression and water suppression, with 1 mm slice thickness and 0.2 mm inter-slice spacing. A total of 6 phases were acquired with a scanning time of 180 seconds per phase, resulting in 108 slices per phase. Post-processing images were reconstructed using Siemens Syngo MR Workplace workstation to generate maximal intensity projection (MIP) images for better visualization of high-intensity areas.

### 2.4 Image analysis

Using the Breast Imaging Reporting and Data System (BI-RADS) by the American College of Radiology (15). All CEM and MRI images were independently analyzed by three radiologists with 10–25 years of experience in breast imaging. To minimize interpretation bias, a delayed crossover blinded reading protocol was implemented. First, each radiologist evaluated the CEM images (low-energy and subtraction images) only and assigned a BI-RADS category. After a washout period of at least four weeks, the same radiologists independently reviewed the MRI images and assigned BI-RADS categories again, without access to their prior CEM interpretations. Throughout the entire image analysis process, all radiologists remained blinded to the final pathological results. Lesions were classified into BI-RADS categories 3, 4A, 4B, 4C, and 5 according to the following criteria:

BI-RADS 3: Likely benign, with a malignancy probability of ≤ 2%. Routine follow-up mammography is recommended, typically within six months.
BI-RADS 4: Malignancy probability between 2-95%, recommending histopathological examination. This category is subdivided into:
4A: Malignancy probability 2-10%, with benign biopsy results permitting routine follow-up in six months.
4B: Malignancy probability 10-50%, requiring combined imaging and pathological evaluation.
4C: Malignancy probability 50-95%, with a high likelihood of malignancy.
BI-RADS 5: Malignancy probability ≥95%, requiring histopathological examination.

### 2.6 Statistical analysis

SPSS 26.0 was used for statistical analysis. Categorical variables were expressed as percentages and absolute values, and continuous variables were presented as mean ± standard deviation. Non-parametric Mann-Whitney U tests were used to assess group differences. Receiver operating characteristic (ROC) analysis was conducted using pathological results as the gold standard, and Z-tests were used to compare the areas under the curve (AUC). McNemar’s test was applied to calculate the sensitivity, specificity, positive predictive value, and negative predictive value of CEM and MRI for detecting benign and malignant breast lesions. Fleiss’ Kappa statistic was used to analyze the agreement among the three radiologists. Kappa values were interpreted as follows: <0.20, slight agreement; 0.21-0.40, fair agreement; 0.41-0.60, moderate agreement; 0.61-0.80, substantial agreement; and >0.81, almost perfect agreement. A p-value of < 0.05 was considered statistically significant.

## 3 Results

### 3.1 Baseline characteristics of the study population

A total of 292 patients were enrolled in this study, with their baseline characteristics summarized in **Table 1**. The cohort had a mean age of 46.9 ± 9.5 years. Notably, more than half (51.0%) of the patients had dense breasts (BI-RADS density categories C or D), and the overwhelming majority (84.6%) of the lesions had been initially assessed as BI-RADS category 4 or 5, indicating high suspicion, before they were referred for CEM and MRI examinations.

**Table 1:** Baseline characteristics of the study population.

Characteristic	Percentage (N = 292)
Age	
Mean ± SD	46.9 ± 9.5
Range	24-78
Breast Density(BI-RADS)	
A(Almost entirely fatty)	28 (9.6%)
B(Scattered fibroglandular)	115 (39.4%)
C(Heterogeneously dense)	121 (41.4%)
D(Extremely dense)	28 (9.6%)
Mode of Detection	
Screening detected	153 (52.4%)
Symptomatic/Palpable	139 (47.6%)
Presenting Symptom	(n = 139)
Palpable mass	118 (84.9%)
Nipple discharge	11 (7.9%)
Focal pain or discomfort	7 (5.0%)
Skin changes	3 (2.2%)
Initial BI-RADS Category	
3	45 (15.4%)
4A	67 (22.9%)
4B	89(30.5%)
4C	61 (20.9%)
5	30(10.3%)

### 3.2 Comparison of diagnostic performance of CEM and MRI for benign and malignant breast lesions

A total of 292 patients underwent paired CEM and MRI examinations, resulting in 301 breast lesions, of which 121 (40.2%) were benign and 180 (59.8%) were malignant, as shown in **Table 2**. According to pathological results, CEM diagnosed 95 benign lesions and 178 malignant lesions, while MRI diagnosed 88 benign lesions and 178 malignant lesions, as shown in **Table 3**.

**Table 2:** Distribution of benign and malignant lesions in the study cohort.

Lesion	Pathology	n(%)
Infiltrating cancer	Invasive ductal carcinoma	143(47.5)
	Invasive lobular carcinoma	4(1.3)
	Papillary and micropapillary carcinoma	2(0.6)
	Adenoid Cystic Carcinoma	1(0.3)
Infiltrating cancer	Ductal carcinoma *in situ*	28(9.3)
	Lobular carcinoma in situ	2(0.6)
Benign lesions	Fibroadenoma	53(17.6)
	Adenopathy	46(15.2)
	Intraductal papilloma	16(5.3)
	Mastitis	2(0.6)
	Galactocele	2(0.6)
	Atypical ductal hyperplasia	2(0.6)

**Table 3:** Comparison of CEM and MRI in diagnosing benign and malignant breast lesions.

Imaging modality	Pathology	
	Benign	Malignant
CEM		
Benign	95	2
Malignant	26	178
MRI		
Benign	88	2
Malignant	33	178

**Figure 2:**
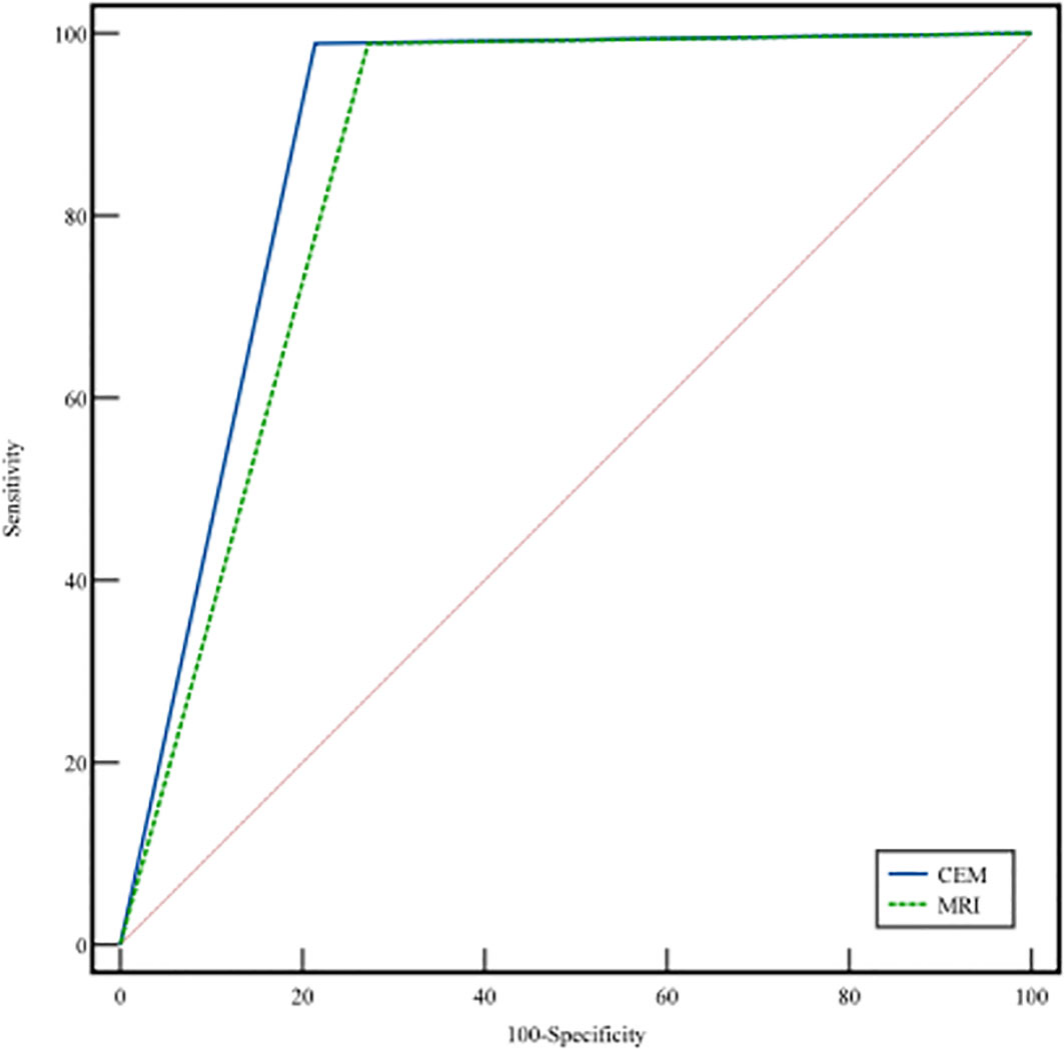
Comparison of ROC curves for CEM (blue line) and MRI (green line).

As shown in **Table 4**, the sensitivity, specificity, accuracy, positive predictive value (PPV), and negative predictive value (NPV) for CEM in diagnosing benign and malignant breast lesions were 98.9%, 78.5%, 90.7%, 87.3%, and 97.9%, respectively. For MRI, the corresponding values were 98.9%, 72.7%, 88.4%, 84.4%, and 97.8%. These results indicate that there is no significant difference in the diagnostic performance of CEM and MRI for breast lesions (p > 0.05). **Figure 2** presents the ROC curves for CEM and MRI. The AUC for CEM and MRI were 0.887 [95% CI: 0.85, 0.92] and 0.858 [95% CI: 0.81, 0.90], respectively (p = 0.400).

**Table 4:** Comparison of diagnostic performance of CEM and MRI in breast benign and malignant lesions.

	Sensitivity(%)	Specificity(%)	Accuracy(%)	PPV(%)	NPV(%)
CEM	98.9	78.5	90.7	87.3	97.9
	[96.0;99.9]	[70.1;85.5]	[86.7;93.7]	[83.0;90.1]	[92.3;99.5]
MRI	98.9	72.7	88.4	84.4	97.8
	[96.0;99.9]	[63.9;80.4]	[84.2;91.8]	[80.1;87.8]	[91.7;99.4]
*p*	1.000	0.369	0.492	0.582	1.000

### 3.3 Comparison of CEM and MRI in classifying breast lesions

**Figure 3:**
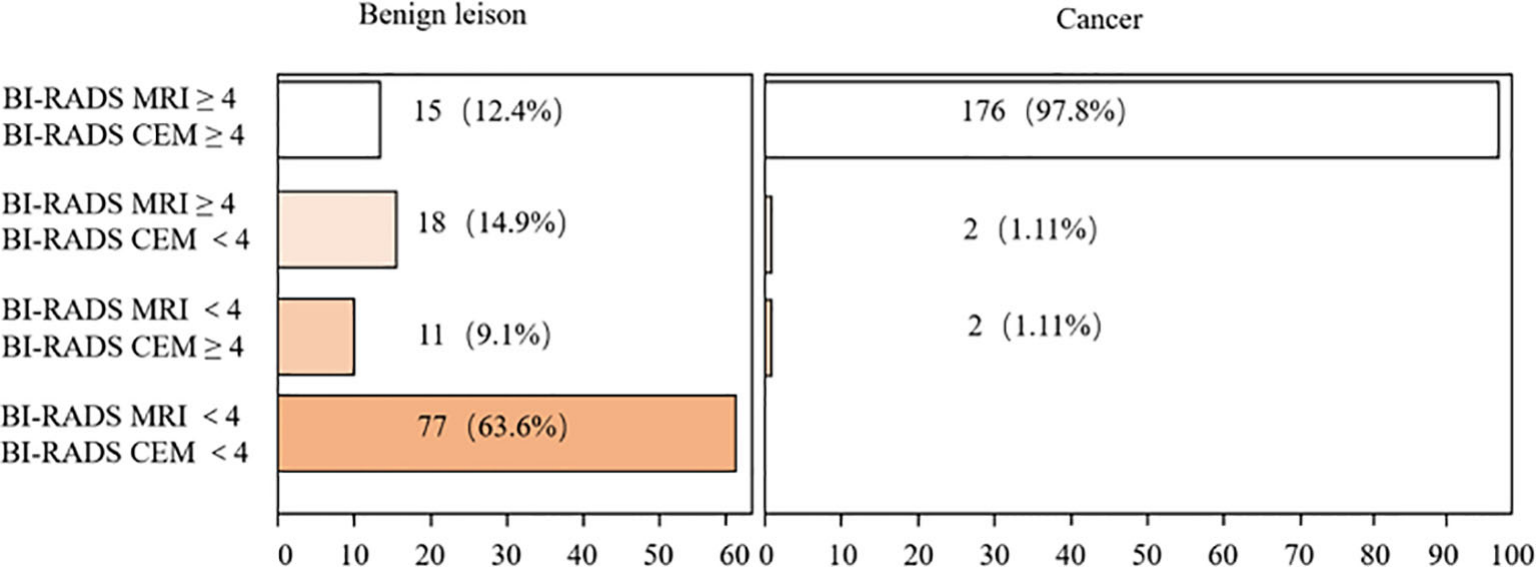
Comparison of BI-RADS classification in MRI and CEM.

As shown in **Table 5**, the diagnostic accuracy of CEM for classifying lesions into categories 3, 4A, 4B, and 5 were 97.9%, 100%, 71.6%, 92%, and 98.6%, respectively. For MRI, the corresponding diagnostic accuracies for categories 3, 4A, 4B, and 5 were 97.8%, 61.9%, 63.5%, 97.8%, and 98.8%. Both imaging modalities demonstrated high accuracy in diagnosing breast lesions across categories 3 to 5. The distribution of BI-RADS classifications for benign and malignant lesions by both modalities is summarized in **Figure 3**.

**Table 5:** Comparison of CEM and MRI BI-RADS classification with pathological results.

BI-RADS	CEM BI-RADS				MG BI-RADS			
	n	Pathology		Accuracy(%)	n	Pathology		Accuracy(%)
		(-)	(+)			(-)	(+)	
3	97	95	2	97.9	90	88	2	97.8
4a	7	0	7	100	21	8	13	61.9
4b	74	21	53	71.6	63	23	40	63.5
4c	50	4	46	92	46	1	45	97.8
5	73	1	72	98.6	81	1	80	98.8

**Figure 4:**
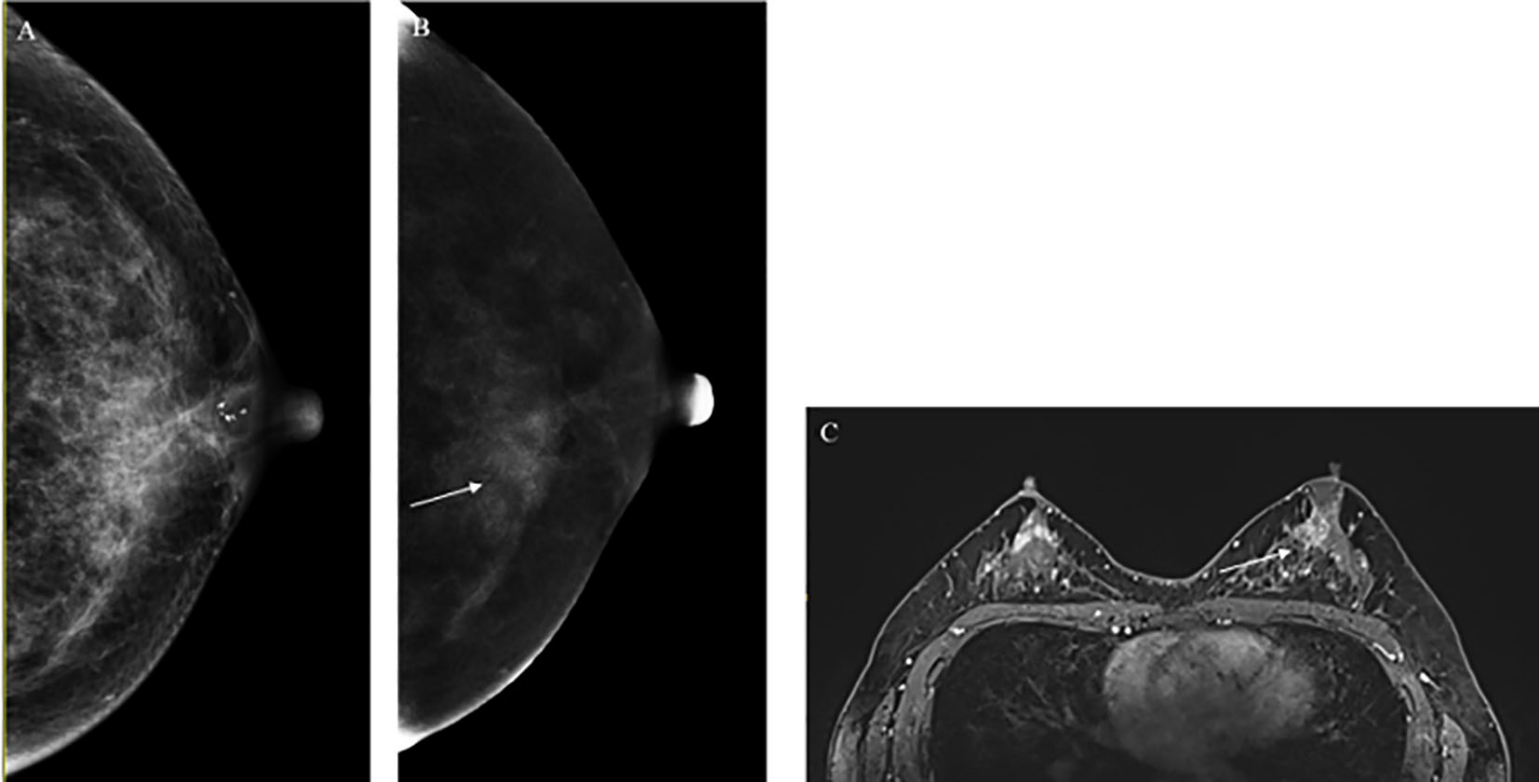
Radiological cases in which both CEM and MRI incorrectly identified benign lesions as malignant. **(A)** CEM CC low-energy image; **(B)** CEM CC subtraction image: A small, patchy isodense lesion (indicated by the white arrow) is observed in the inner upper quadrant of the left breast, with irregular edges and enhancement upon contrast administration; **(C)** MRI T1-weighted enhanced image: A planar, irregularly enhanced focus is seen in the left nipple region. Both imaging modalities suggest a tumor-like lesion, classified as BI-RADS 4C. Pathological findings revealed a papillary tumor with ductal epithelial hyperplasia.

For benign lesions, 63.6% of the lesions had a consistent and correct BI-RADS classification in both MRI and CEM, as compared to the histopathological results. However, 37.3% of lesions were classified as BI-RADS 4 or higher by MRI, while CEM incorrectly classified 21.5% of the lesions as BI-RADS 4 or higher. **Figure 4** illustrates cases where both CEM and MRI mistakenly identified benign lesions as malignant.

**Figure 5:**
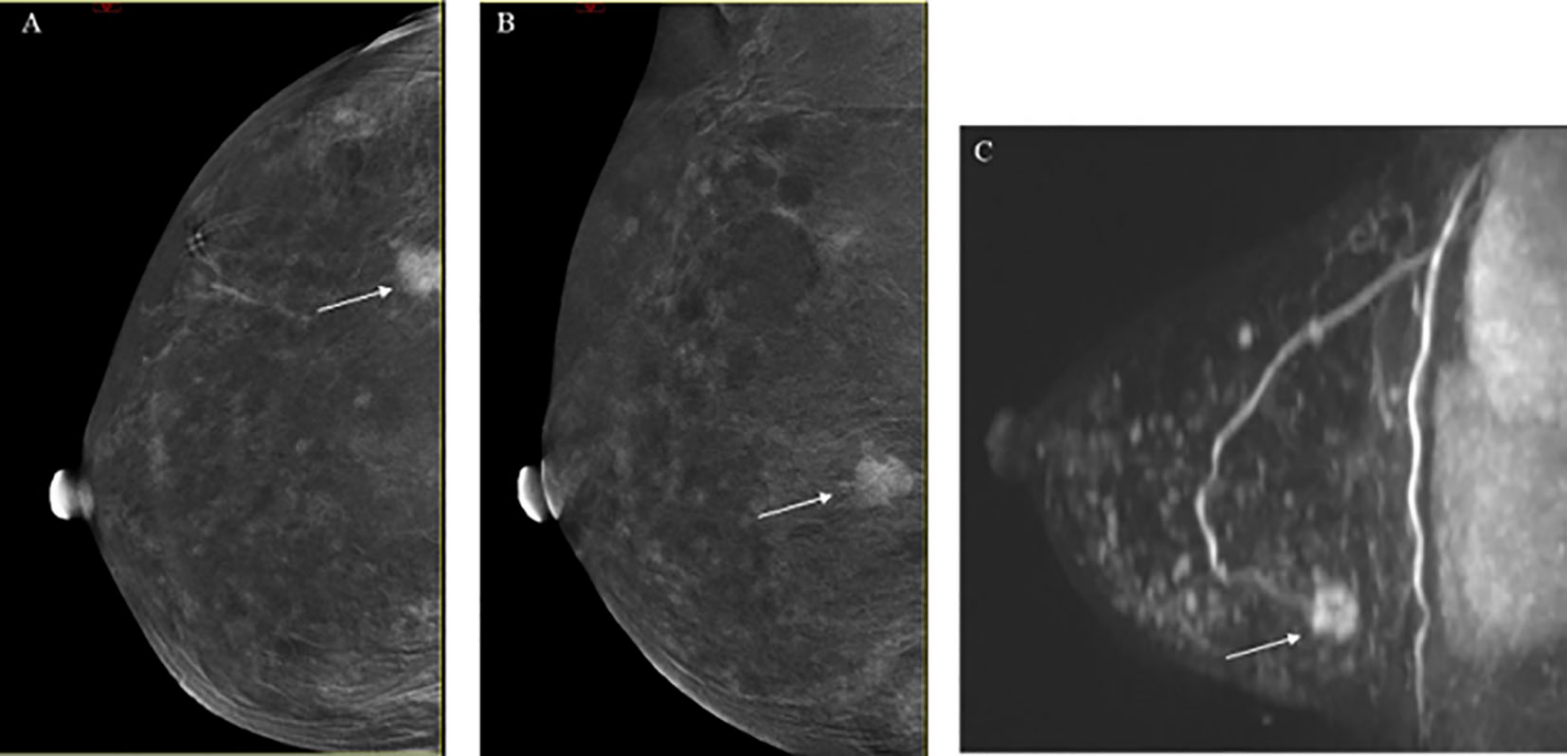
Correct diagnosis of malignant cases by CEM and MRI imaging. **(A)** CEM CC view **(B)** CEM MLO view: A slightly high-density mass (indicated by the white arrow) is visible in the lower outer posterior part of the right breast. The mass is lobulated with spiculated borders and shows marked enhancement. This corresponds to **(C)** the MRI MIP image, where a relatively large blood vessel is observed entering the lesion. The pathological result is invasive ductal carcinoma.

For malignant lesions, 97.8% of the lesions were correctly classified by both imaging techniques. A small percentage, 1.11%, were misclassified as benign by either MRI or CEM. **Figure 5** shows cases where both CEM and MRI correctly diagnosed malignant lesions.

### 3.4 Inter-reader agreement

The three radiologists demonstrated almost perfect agreement in differentiating benign from malignant lesions based on BI-RADS categories. For CEM, Fleiss’ kappa was 0.84 (95% CI: 0.79-0.91); for MRI, Fleiss’ kappa was 0.86 (95% CI: 0.82-0.92).

## 4 Discussion

Breast MRI has a high sensitivity for breast cancer detection and, through multiparametric evaluation, can effectively differentiate between benign and malignant breast lesions. MRI is superior to other imaging modalities in evaluating tumor size, detecting other lesions in the same or opposite breast, and assessing the response to neoadjuvant chemotherapy (16). However, due to its high cost, time consumption, and potential to increase patient anxiety, MRI may delay the overall treatment plan and pose challenges for clinical communication between doctors and patients.

Contrast-enhanced mammography (CEM) is an emerging technology that uses iodinated contrast agents to detect tumor vasculature. The U.S. Food and Drug Administration (FDA) officially approved CEM in 2011 as an adjunct to mammography and/or ultrasound for breast cancer evaluation (17). CEM enhances the diagnostic performance of traditional mammography. Research by Lobbes et al. showed that compared with mammography, CEM sensitivity increased to 100.0% (from 96.9%), specificity increased to 87.7% (from 42.0%), PPV increased to 76.2% (from 39.7%), NPV increased to 100% (from 97.1%), and AUC improved from 0.779 to 0.976, with all differences being statistically significant (18). Dromain’s study also indicated that CEM has superior diagnostic accuracy compared to mammography and mammography combined with ultrasound (14). Compared to the subtraction images alone, the combination of low-energy images showed higher sensitivity (95% vs. 94%, p < 0.001) and specificity (81% vs. 71%, P = 0.03). Additionally, for patients with dense breasts, CEM had a sensitivity of 95% and specificity of 78% (19).

This study found that the diagnostic performance parameters for CEM were sensitivity 98.9%, specificity 78.5%, accuracy 90.7%, PPV 87.3%, and NPV 97.9%. No statistically significant differences were found between CEM and MRI for diagnosing benign and malignant breast lesions in terms of sensitivity (98.9% vs. 98.9%), specificity (78.5% vs. 72.7%), accuracy (90.7% vs. 88.4%), positive predictive value (87.3% vs. 84.4%), and negative predictive value (97.9% vs. 97.8%) (p > 0.05). The AUC for CEM and MRI were 0.887 and 0.858, respectively (p = 0.400). Previous studies have also shown that CEM and MRI have similar diagnostic efficacy. In a study by Stephanie et al., which included 52 women and 120 breast lesions, the most common imaging manifestation for index tumors on MRI was a mass, while CEM most commonly showed mass-like enhancement and non-mass-like enhancement. For 11 cases of secondary cancer, CEM detected all 11, while MRI detected 10. In terms of diagnostic performance, CEM and MRI had similar sensitivity (94% vs. 99%)—similar to the study by Jochelson et al (20, 21). A prospective multicenter study involving 178 patients found that CEM alone and CEM combined with mammography had AUCs of 0.84 and 0.83, respectively, both higher than mammography (0.76), but without a significant difference compared to MRI (0.85). Wang et al. included 68 patients who underwent both MRI and CEM for breast lesions and found that CEM had a sensitivity of 95.8% and specificity of 65.5%, while MRI had a sensitivity of 93.8% and specificity of 82.8%. The Bland-Altman plot showed that the average tumor measurement difference between CEM and MRI was 0.7mm, with CEM showing better correlation with pathological results than MRI, suggesting that CEM may offer superior diagnostic efficacy in terms of sensitivity and tumor size (22). Jakubowicz et al. found that CEM’s diagnostic efficacy was slightly better than MRI, with CEM showing higher sensitivity (100% vs. 93%) and accuracy (79% vs. 73%), and the AUC based on BI-RADS was 0.83 for CEM and 0.84 for MRI (23). Meta-analyses have shown that MRI has higher sensitivity than CEM (97% vs. 91%) but lower specificity (69% vs. 74%). The studies also found that iodine concentration is positively correlated with CEM sensitivity (P = 0.04) and negatively correlated with specificity (P < 0.001) (24). A recent meta-analysis reported that the sensitivity of CEM was 96%, and MRI had a sensitivity of 97%, with both having a specificity of 77%. Although MRI’s diagnostic odds ratio (DOR) was 122.9, higher than CEM’s DOR of 79.5, the researchers noted that current scientific evidence is limited, and it is premature to consider CEM as a non-viable alternative to MRI (25). Another meta-analysis showed that both CEM and MRI have high sensitivity (0.96), but CEM outperforms MRI in detecting index lesions, distinguishing suspicious lesions, and evaluating dense breast tissue, suggesting that CEM could serve as an alternative imaging method to MRI (26). Sumkin et al. found that CEM and MRI had sensitivity rates of 93% and 91%, respectively, for detecting malignant tumors, but MRI identified more suspicious benign lesions than CEM, leading to a lower positive predictive value for additional biopsies (27). Tradivel et al. found that CEM changed the surgical and diagnostic strategy for 21% of patients in breast cancer staging, resolving suspicious findings in routine breast imaging or detecting recurrences (12).

CEM provides enhanced information for diagnosing breast microcalcifications. For suspicious microcalcifications, CEM enhancement of the calcifications may suggest underlying cancer, with an overall sensitivity of 88.89%, specificity of 86.56%, and accuracy of 87.24% (28). However, some studies have indicated that CEM is not statistically superior to full-field digital mammography (FFDM) in guiding surgical decisions based on calcification information (29). Asymmetry with enhancement is highly indicative of malignancy, while asymmetry without enhancement suggests a higher likelihood of benign lesions (30). CEM’s dynamic curve can also distinguish between benign and malignant lesions. Rong et al. found significant differences in the enhancement patterns of benign and breast cancer lesions (P < 0.001), with the kappa coefficient for the dynamic curves of CEM and MRI being 0.752 (p < 0.001) (31). However, it is important to note that some benign lesions may also show enhancement on CEM, with the most common being fibroadenomas, papillomas, and proliferative changes (32).

This study provides robust evidence from a Chinese population for the comparable diagnostic performance between CEM and MRI in evaluating breast lesions, based on a head-to-head comparison of 292 patients. Notably, our research offers several novel insights. First, compared with many prior studies, we enrolled a substantial proportion of patients with dense breasts (51.0%), and this study confirms the excellent performance of CEM in this diagnostically challenging population, where its specificity was even slightly higher than that of MRI. This adds significant support for the wider application of CEM in Asian women, a specific demographic often characterized by dense breast tissue. Second, our detailed analysis of BI-RADS category 4 subcategories revealed that CEM demonstrated superior accuracy (100%) in discriminating low-suspicion lesions (category 4A), a finding of direct relevance to clinical decision-making. Finally, in an era of increasing focus on healthcare resources, our findings strengthen the position of CEM as a viable alternative to MRI, offering comparable diagnostic performance while being more cost-effective and accessible, which holds significant practical implications for optimizing resource allocation and improving the efficiency of diagnostic pathways.

Our study has several limitations. Firstly, its single-center, retrospective design may introduce selection bias. Future prospective, multi-center studies are needed to validate our conclusions. Second of all, although we implemented a delayed crossover blinded reading protocol with excellent inter-reader agreement (Kappa > 0.85), radiologists might still infer some clinical information from the anatomical context of the images. Therefore, this remains a single-blind design where potential interpretation bias persists. In addition, while pathological confirmation served as the gold standard for accurately assessing sensitivity, this study could not determine the true false-negative rates of both modalities through long-term imaging follow-up. Future prospective studies should incorporate regular follow-up (e.g., at 12 months) for individuals with negative imaging findings, which is crucial for establishing the interval cancer rates and ultimate false-negative rates in a screening context. Finally, although we conducted preliminary explorations through subgroup analyses based on breast density and age, other factors that might influence diagnostic performance (such as specific lesion morphology and enhancement patterns) were not thoroughly investigated. Studies with larger sample sizes are warranted to further identify specific patient subgroups that would benefit most from either CEM or MRI.

## 5 Conclusion

In conclusion, CEM is an emerging imaging technology that offers several advantages, including lower cost, shorter examination time, and better patient tolerance. It is also capable of accurately detecting and diagnosing breast lesions. Similar to MRI, CEM demonstrates high sensitivity and accuracy, with its specificity even surpassing that of MRI. Future multi-center studies with larger patient populations are needed to further clarify the diagnostic benefits of CEM in breast cancer detection.

## Data Availability

The raw data supporting the conclusions of this article will be made available by the authors, without undue reservation.
