# Correction: Comparison of diagnostic performance between contrast-enhanced mammography and breast magnetic resonance imaging

**DOI:** 10.3389/fonc.2026.1829116

**Published:** 2026-03-23

**Authors:** Ying Yang, Xiangying Yang, Fan Yang

**Affiliations:** Department of Radiology, Union Hospital, Tongji Medical College, Huazhong University of Science and Technology, Wuhan, Hubei, China

**Keywords:** breast cancer, contrast-enhanced mammography, diagnostic performance, magnetic resonance imaging, mammography

Affiliation “Department of Radiology, Union Hospital, Tongji Medical College, Huazhong University of Science and Technology, Wuhan, Hubei Province, China” was erroneously given as “Wuhan Union Hospital, Tongji Medical College, Huazhong University of Science and Technology, Wuhan, China”.

The original version of this article has been updated.

